# Design of the Maternal Website EMAeHealth That Supports Decision-Making During Pregnancy and in the Postpartum Period: Collaborative Action Research Study

**DOI:** 10.2196/28855

**Published:** 2021-08-09

**Authors:** Isabel Artieta-Pinedo, Carmen Paz-Pascual, Paola Bully, Maite Espinosa, EmaQ Group

**Affiliations:** 1 Osakidetza-Basque Health Service Biocruces-Bizkaia Health Research Institute Osi Barakaldo-Sestao Barakaldo Spain; 2 Paola Bully Methodological and Statistical Consulting Sopuerta Spain; 3 Biocruces-Bizkaia Health Research Institute Barakaldo Spain

**Keywords:** prenatal education, women, patient decision aid, decision-making, clinical decision support systems, action research and pregnancy, implementation science, health service needs and demands

## Abstract

**Background:**

Despite the benefit maternal education has for women, it needs new tools to increase its effectiveness and scope, in tune with the needs of current users.

**Objective:**

We attempted to develop a multifunctional personalized eHealth platform aimed at the self-management of health in relation to maternity, which can be considered a flexible and adaptable maternal education tool.

**Methods:**

The International Patient Decision Aid Standards (IPDAS) were applied. A website prototype was developed for implementation in the public health system using a collaborative action research process, in which experts and patients participate, with qualitative research techniques, as well as focus groups, prioritization, and consensus techniques.

**Results:**

We have proposed a website that includes (1) systematically updated information related to clinical practice guidelines, (2) interaction between peers and users/professionals, (3) instruments for self-assessment of health needs as a basis for working on counseling, agreement on actions, help in the search for resources, support in decision-making, and monitoring and evaluation of results, and (4) access for women to their clinical data and the option of sharing the data with other health agents. These components, with different access requirements, would be reviewed through iterative cycles depending on the frequency and effectiveness resulting from their use and would be accessible from any digital device.

**Conclusions:**

A website that supports maternal education should contain not only information, but also resources for individual attention and social support. Its usefulness for the health and satisfaction of women should be evaluated in various different environments.

## Introduction

### Background

Permanent and sometimes abrupt changes in our societies are undeniable facts, and changes in health, society, and technology often come quickly and simultaneously. Health care tries to respond to increased demand and the current needs of users with the new resources available. In recent years, the examples of services that use new technologies in the care of patients with chronic pathology or multimorbidity have multiplied, and telemedicine has a widely understood potential for training and support in shared decision-making [1,2], using tools including video consultation, mobile messaging, and expert systems that issue responses from clinical practice guides or available resources. Sometimes they are accessible from platforms that are outside the health system but very widespread among the population, such as Facebook [3].

Pregnant and postpartum women represent a demographic that is especially in need of support. The transition to motherhood involves a change of identity, social role, and activity from the known to a new reality [4,5]. In this process, they feel great pressure to do well, which can ultimately reduce their well-being [6]. Stress during pregnancy or in the postpartum period generates physical and psychological responses, which can ultimately lead to disorders such as depression, anxiety, and emotional distress [7]. The effort of professionals to generate preparation tools for childbirth and the promotion of physical and psychological health are common [8,9]. However, there is also agreement on the need to update these tools [7,10]. The use of e-technologies offers advantages, such as accessibility at any time and place, and for a very large population, information can be gathered in a personalized way and the user can review the resource as much as desired.

Women of reproductive age, and more specifically, those in the stage of maternity, represent a population group that is especially likely to benefit from the use of these new technologies. They systematically use the internet as a source of information, including information about their health [11], and frequently base their decisions on the information obtained [12,13].

Previous experiences with the use of telemedicine during pregnancy have given positive results in the prevention of pre-eclampsia [14] and in the management of diabetes [15]. In the postpartum period, online communication between peers has been associated with lower levels of anxiety and feelings of loneliness [16] and attention through a website [17] or a mobile app [18], with a higher perception of self-efficacy, psychological well-being, and satisfaction with the care received. The availability of computer-based educational programs has also been shown to be associated with greater self-efficacy and a more positive attitude toward breastfeeding [19]. However, the determinants of its effectiveness remain to be explored, since the results vary depending on the behavior studied [20], the time of measurement [21], or the population studied [22].

It seems necessary, and indeed urgent, to have a web tool to support decision-making for the maternity stage, which is reliable [23], personalized [24], and based on the women themselves and the initiatives of professionals [25]. The Medical Research Council provides a theoretical framework for the development of this complex intervention [26] and recommends actions that facilitate implementation in the real world. Therefore, it was decided that the website should include people who teach and receive maternal education from the beginning, and a Participatory Action Research study, based on theories of behavioral change, was used. The integrated model proposed by Fishbein et al [27] was considered to be the most appropriate since it takes into account the influence of attitudes, self-confidence, sociodemographic variables, and social norms on the intention to act and ultimately on the achievement of healthy habits. The influence of these variables, in addition, would be modified by barriers and facilitators in the environment. This theoretical model has been successfully applied in the study of sexual behavior [28] and has been used in similar studies to prevent excessive weight gain during pregnancy [29].

The development of this tool must follow a strategy that takes into account the resources, needs, and characteristics of the target population, and for this development, a collaborative environment should be created, involving the population, putting it into practice, implementing it, and evaluating the process through iterative cycles of continuous improvement [30].

### Objective

We attempted to develop a multifunctional and personalized eHealth platform, bringing together patients and professionals, which allows them to attend and monitor their health needs and take informed decisions during pregnancy, childbirth, and parenting. Subsequently, its clinical effectiveness and its implementation in routine conditions will be evaluated (usability and acceptability by users and professionals, and impact on the health system). This article focuses on describing the design and development of the tool.

## Methods

### Procedure for the Development of the EMAeHealth Tool

**Figure 1:**
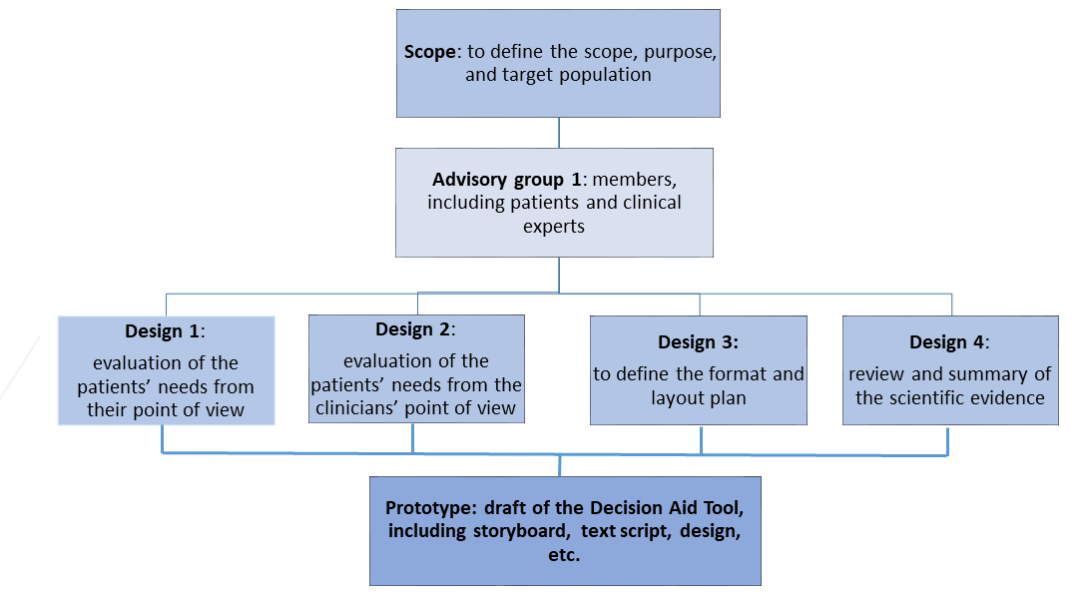
Development process of a decision aid tool. Adapted from the report by Perestelo-Pérez L et al [31].

For the development of the “EMAeHealth” tool, the International Patient Decision Aid Standards (IPDAS) were followed (Figure 1) [31]. The shared decision-making (SDM) model is defined as an approach in which health care professionals and patients make decisions together using the best available evidence. The SDM model emphasizes respect for the patient’s autonomy and promotes training so that the patient is involved in the decision-making process.

The study was conducted in Basque Country, a region in the north of Spain with a population of 1.2 million. This region has a public health service, which is free at the point of use, and is universally and readily accessible, with local centers that provide care for people living in the surrounding geographical area. During pregnancy, women are monitored via alternating appointments with their primary care midwife and gynecologist, and in the last trimester, they are invited to attend antenatal education sessions run by one of the midwives at the health center.

### Scope

We propose a flexible maternal education tool that will be adapted and adaptable to each population group, offered universally by the public health system, and that supports women continuously throughout the maternity process.

### Advisory Group

A multidisciplinary research team was formed of hospital and primary care midwives, pediatricians, psychologists, and experts in methodology. The process of pregnancy and parenting is so common that the team could hardly distance itself from their own experiences as mothers, which provided a “patient” vision.

### Design 1

A qualitative study (focus groups) was carried out following the guidelines of Krueger and Casey [32]. Women who went to see their midwife were recruited consecutively. Two groups were formed (pregnant women and postpartum women), and they were given a questionnaire that allowed each of the two groups to be divided according to income level and education. Finally, 31 women participated in four groups that were homogeneous in terms of parity and socioeconomic status. Each study participant was given the study fact sheet along with an informed consent form, and all of them agreed to audio recordings of the meetings. For the collection of information, three experts in qualitative methods designed a script related to the topics to be discussed (perceived needs regarding pregnancy care and maternal education), using the thematic content analysis method. Three researchers independently read the transcripts of the sessions and arranged the information into the topics to be discussed. They assigned codes to the text segments and regrouped them into categories using ATLAS.ti software (Scientific Software Development GmbH). With these categories and subcategories, and the relationships between them, a conceptual structure was constructed by each of the analysts. It was later triangulated and compared again with the text to give the final results [33].

### Design 2

The advisory group was joined by other professionals, including a gynecologist, two pediatric nurses, experts in breastfeeding and health education, epidemiologists, psychologists, sociologists, and experts in qualitative research. The selection of these people was based on the opinion of the research team and on their work experience and previous interest and participation in research work. Finally, a group of nine women and two men was formed (age between 30 and 57 years), with health care experience ranging from 5 to 30 years.

Nominal group techniques were used in the sessions. After a study and individual analysis work, each team member had 5 minutes to explain the main health needs of women during pregnancy and the first year postpartum. After the round of presentations, time was devoted to identifying common ideas. In those in which there was a discrepancy, explanation and defense was allowed, and they were finally ranked by voting [34].

### Design 3

In order to encourage participation and implementation of the tool, a meeting was scheduled, to which all active midwives in the health centers of Bizkaia and in the hospitals of Cruces and Basurto were invited. This meeting was attended by 25% of the total 20 midwives. They were shown the results obtained in the previous steps and asked to choose the actions they considered most feasible and relevant from all the proposals for a new framework of continuous personalized maternal education [34].

### Design 4

A descriptive study was carried out. Using the most widely used internet search engines in our region (Google, Yahoo, and Bing), the first 25 web pages that appeared when entering words, such as pregnancy, childbirth, postpartum, and breastfeeding, in Spanish and English were selected.

Each of these websites was evaluated by the midwives participating in the study using the LIDA questionnaire, version 1.2 [35] as a measure of reliability, accessibility, and usability, since it is a validated questionnaire that considers the characteristics of the author’s reliability, conflicts of interest, references, and relevance common in other studies [36]. Each of the websites received a score on this questionnaire.

Throughout the process, care was taken to ensure the trustworthiness of the data. Several facts give credibility and reliability to the data obtained, including its origin in a team made up of health care professionals with high involvement in their fields and observations over the years, the high degree of agreement between professionals from different fields (pediatrics, gynecology, nursing, and hospital and primary care midwifery), and the transferability that has been sought through representativeness in both women in the focus groups and the midwives who were asked to select the most feasible and priority interventions. We think that the duration, the coherence between the different groups and stages, the congruence with the bibliography, and the inclusion of different types of professionals and women allowed us to reasonably assume the credibility, transferability, dependability, and confirmability of our data.

Both the design of the website and its subsequent evaluation have received a favorable report from the Ethics Committee for Clinical Research of the Basque Country (PI2012072 and PI20200044).

## Results

### Design 1

It was seen that the focus of women’s worries were different based on the stages of pregnancy/postpartum. In early pregnancy, women’s main concern was for “everything to go well.” As the pregnancy progressed, they needed emotional support and wanted to feel confident and be self-reliant to face their fears of the birth and care of their children. The needs expressed by women were as follows: accurate information that was accessible and suited to their specific life moments; flexible maternal education programs in terms of schedule and content; and greater participation of partners. All of them had a positive opinion of our health system and the role of midwives, although they would like more support after giving birth. Puerperal women reported “excessive pressure” in favor of breastfeeding despite its difficulty [33].

A website allows permanent accessibility and continuity from the beginning of pregnancy to 1 year after delivery, so it would respond to several of the needs expressed. This website would require rigorous stable information based on clinical practice guidelines. It should also allow interaction, in order to address the specific doubts of each moment of the motherhood process (for the woman or her partner).

### Design 2

After seven sessions, the group of experts proposed three moments in which important changes in women’s health needs can be seen as follows: at the beginning of pregnancy, at the onset of childbirth, and in the first months after childbirth. Three interactive exploratory tools were proposed for identifying potential difficulties, establishing peer-to-peer and health care communication, and negotiating possible solutions to specific needs. Personalized intervention is key, because a woman’s interest and resources vary depending on personal characteristics such as age, race, parity, and pregnancy evolution.

The care should support the identification of specific needs, and guide the advice and support for the achievement of personal goals in areas such as choice of the type of delivery, breastfeeding, or parenting [36].

The proposed website should, therefore, include self-assessment tools, preferably appropriate to the three key moments, and would provide possible answers for the need felt, allowing the woman’s agreement to be checked and the case to be followed up. For example, answers could be given to doubts about a healthy diet and proper weight gain, or providing resources for the proper management of childbirth anxiety.

### Design 3

Casting a vote, all the participant midwives considered that a web format that would allow communication with the women would be the most useful, would offer the most possibilities of meeting the needs expressed by the women regarding continued and accessible care, and would facilitate the solution of occasional problems. They also considered it very necessary to have a channel of communication between the women who live in the same area and share a health system or similar pregnancy protocols, to facilitate the formation of social networks and the solution of frequently asked questions. They believe that this forum could be moderated by a professional.

### Design 4

The quality of information available on the internet regarding pregnancy, childbirth, and the postpartum period in general was moderate, poor, or very poor, with rare references to the source of information. Reliability was higher on websites belonging to public bodies, universities, or health companies than on websites belonging to commercial companies, and was higher among all of them than on websites of individuals or small private groups.

It was found that the higher quality websites were up-to-date, with information based on scientific evidence, frequently including videos and personal experiences of other women, and the possibility of interacting with peers and health professionals [36].

### “EMAeHealth” Prototype

**Figure 2:**
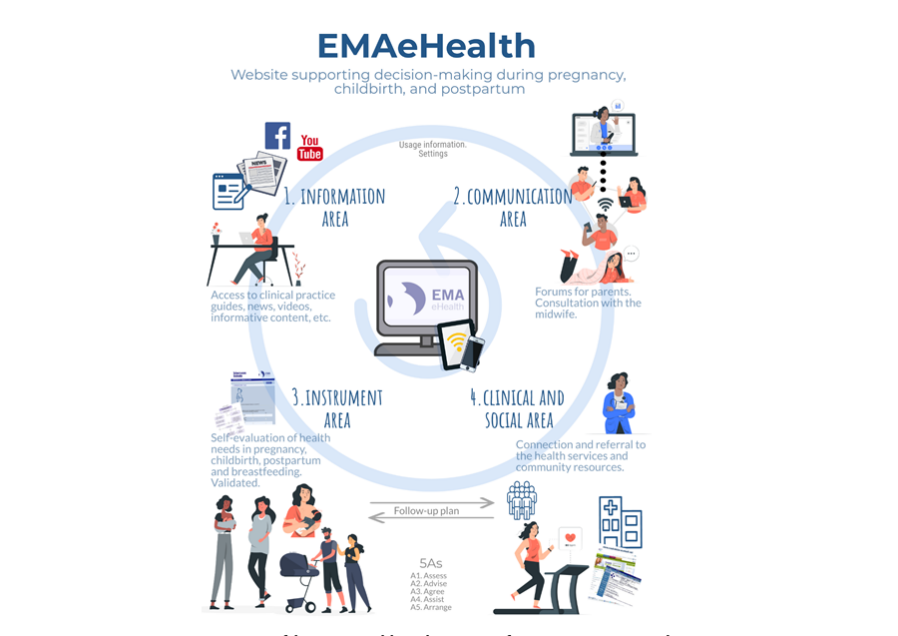
Diagram of the EMAeHealth tool prototype for supporting women during pregnancy, chidbirth, postpartum, and breastfeeding/parenting. There are four main areas.

As a result of the previous analysis and reflections, a prototype named the “EMAeHealth” web portal (Figure 2) was developed. The name stands for “maternal education” in Spanish (educación maternal). It includes the following *functionalities*: (1) help women to self-evaluate and prioritize their health needs related to pregnancy, childbirth, puerperium, lactation, and child care, identifying people and populations at risk of physical, mental, or social complications; (2) promote training and self-management, establish decision-making support systems, and serve as a guide in identifying available resources; and (3) allow access to content from multiple devices and operating systems, and store the data safely.

**Figure 3:**
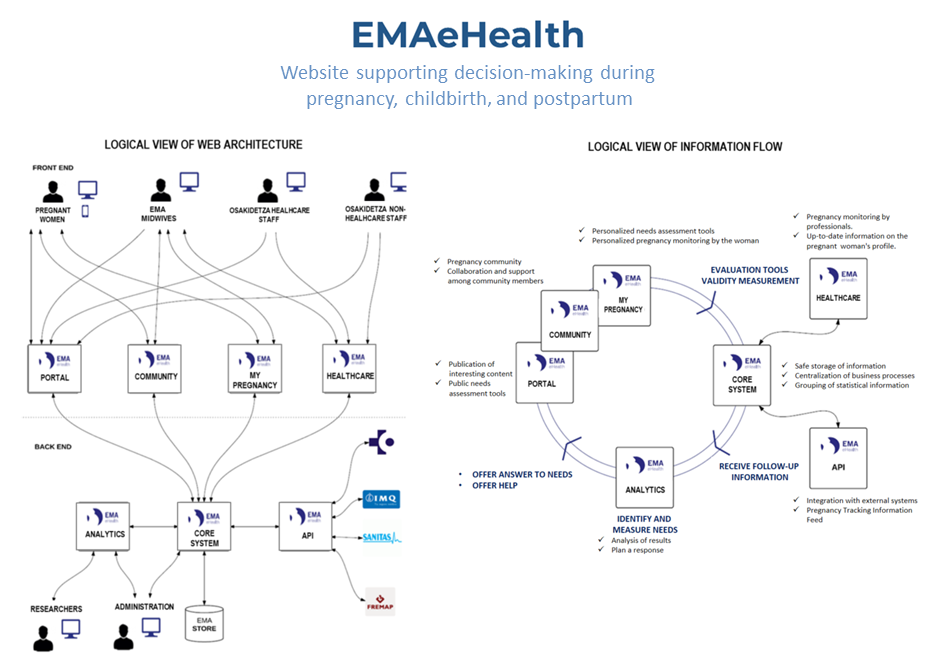
Architecture and information flow of the EMAeHealth website.

Experts in software engineering propose a *structure* (Figure 3) in which, starting from a home page with the general menu, the different contents are distributed in a modular structure related to the different needs and offers, and with different degrees of accessibility based on the identity of the user (patient, professional, and health system). An initial module would give access to publicly available information and to various self-assessment tools, which would not offer any further resources (EMAportal). A second module would allow access to both peer communication forums and resources available in response to self-assessment, for which the woman would have to identify herself as a member of the group (EMAcommunity). A third module, which is more complex, would allow access to health data and the monitoring of care, for which a login would be needed to guarantee confidentiality (EMAhealthcare). The structure would allow professionals and administrators to get statistics on its use and health outcomes, enabling the modification, elimination, or enhancement of different areas.

**Figure 4:**
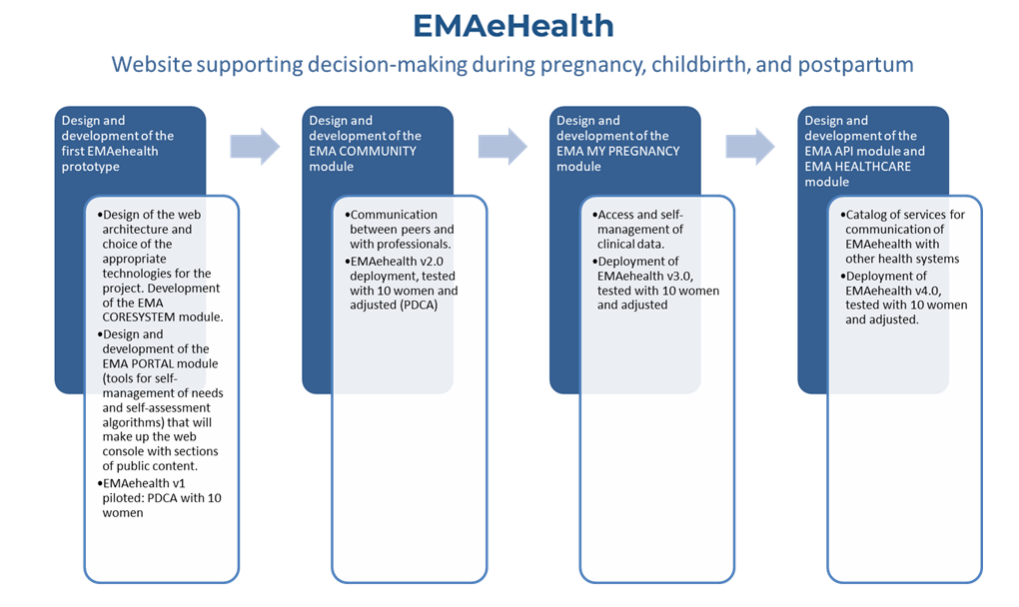
Flowchart of the development of the EMAeHealth tool up to its clinical application.

Functionalities and applications would be added to this initial structure based on the feedback received (Figure 4).

The platform contains a file or data repository for monitoring information, with a sufficient guarantee of storage security (EMAcore), which allows the transmission of this data between servers of different information systems and their connection between several systems (the Basque Health System *Osakidetza* and other health and social systems) (EMAApi). The complete development of this website would involve the participation of the organization where the woman is registered for the pregnancy, and therefore, the most personal data and interactions should be stored along with the rest of the medical history, thus ensuring confidentiality.

Regarding the *content*, the EMAeHealth portal contemplates interaction with the woman and her partner at various levels as presented below.

#### Information Area

**Figure 5:**
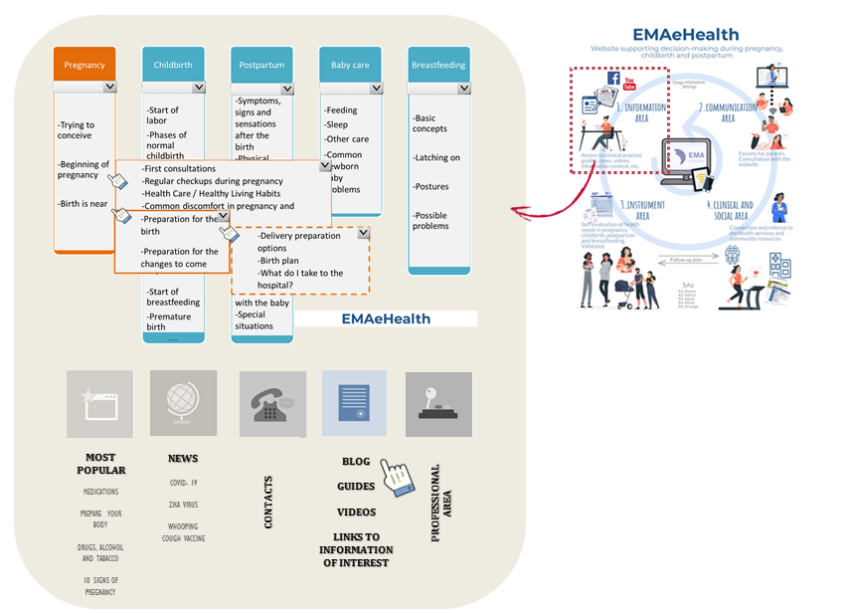
Contents in the EMAeHealth information area. Information based on available clinical evidence, selected and reviewed by professionals.

This is an area open to the public that will offer information based on evidence and be constantly updated by health service professionals (Figure 5). The website will inform users about the maternity process, including the desire for pregnancy; first, second, and third trimesters; labor and delivery; immediate and late postpartum; and breastfeeding. It would also include care of the newborn, paying special attention to the vaccination calendar during the baby’s first year of life by sending reminders about the dates and following up on the correct vaccination. In each section, the physiology of the process, healthy lifestyle, possible health problems, common remedies, and available resources will be described.

Current news will also be posted such as changes in legislation related to the process and the effects of the Zika virus and COVID-19. Applications, such as calculators, estimates of appropriate weight, an exercise calendar, and healthy diets will be offered.

It will be presented in multiple formats and support blogs, videos, interviews, articles, and data suitable for different types of population. The portal will have a data monitoring and management system that will allow the research team to collect browsing statistics on the different sections and adjust the content if necessary.

#### Communication Area

This is an area that will require user login and will allow the female user to contact other women from a close environment and in a situation similar to her situation, through forums or conversations, or consult with her health care professional. Peer interaction is one of the most highly valued functions among users of digital tools [37,38], and it has been shown to increase postpartum mental well-being [18]. The regular use of social networks to not only receive but also contribute knowledge can favor a greater connection to the tool and therefore greater access to information based on evidence and greater possibility of interaction with the health care professional [38]. A professional will moderate the tool on a rotating basis and answer questions. There will be an administrator who can block anyone who distorts the content or alters relationships. In a project like this, part of the work of midwives would be focused on non-face-to-face interventions, so work plans should also be adapted to this. Primary care midwives currently provide much of the service through prearranged appointments, and this time could be split between face-to-face consultations and online interaction.

On the other hand, the areas that need identification will only be available to the woman during pregnancy and the first year postpartum, or until the end of the pregnancy in the case of abortion. In this way, the women included share close experiences in time and space, which would predictably make the contact more useful. Access would be reactivated for each pregnancy for a similar period.

#### Health Self-Management Area

**Figure 6:**
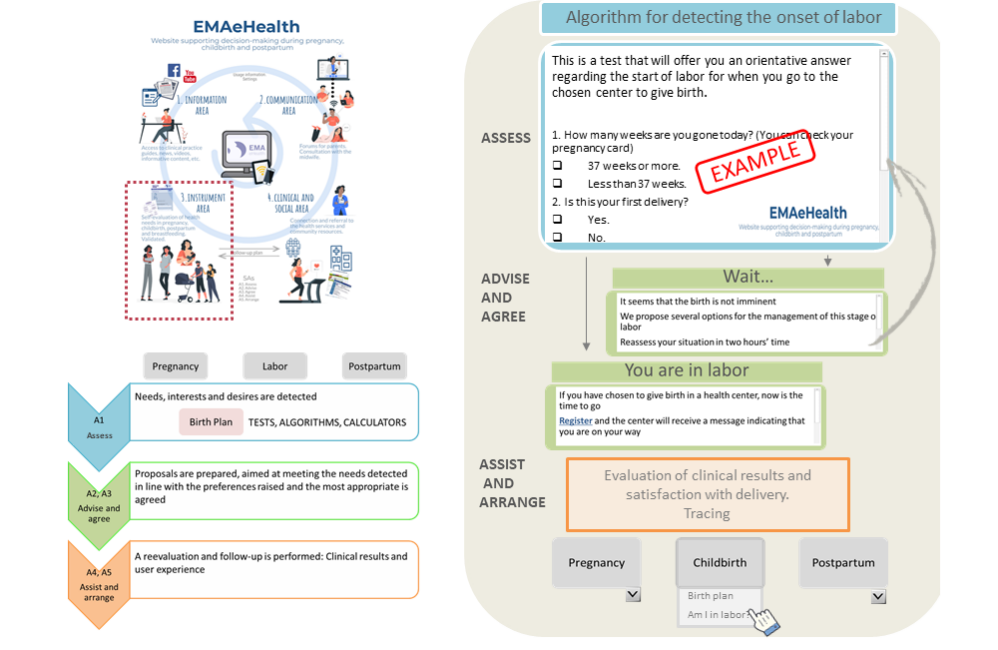
Contents in the EMAeHealth self-assessment instruments area. The algorithm is currently under development and validation, and thus, it may be subject to change.

This area has valid and reliable *self-assessment instruments* to check or reflect on one’s own health needs (Figure 6). It consists of scales and questionnaires that cover preferences, personality traits, culture, or beliefs that can determine a decision and the satisfaction obtained with it. As suggested by the group of experts, these self-assessment tools could be presented at least at three key moments as follows: start of pregnancy, onset of birth, and postpartum period. In this space, we would work directly with individual health needs, applying the following five As: assess (self-assessment and prioritization), advise (training/support), agree (planning actions), assist (guides to available resources), and arrange (carrying out monitoring and evaluating the results). Following this procedure, the woman responds to a validated questionnaire on her needs and preferences related to childbirth, and depending on the results, the most suitable alternatives and their availability are offered. Once the woman selects her favorite, the information on how to access it is provided, and follow-up and feedback on the result achieved are agreed. This process will often be mixed, combining the responses from the digital tool and the midwife who attends to the woman.

The same strategy will be followed with all those topics that, based on the information collected on the web, demonstrate their relevance and interest for the user population (eg, food, newborn care, and pelvic floor rehabilitation). The validation process of the self-assessment tools initially included is currently being carried out with the support of the Basque Government (Basque Government Health Department; grant number: 2018111087).

#### Clinical Data Area

This area will require user login, and the woman will be able to see her clinical data, add the most recent items, and share them with other professionals if she wishes.

The safe storage of the data and feedback will allow the information presented to be analyzed and adjusted. The website will provide the team with the necessary interface to be able to include, validate, and evaluate new elements in each area (EMAAnalysis). Finally, the website should be accessible from any digital device (eg, phone, tablet, computer, and smart TV) and for any company or organization (eg, health and social) that the woman wishes to use and allow access, naturally with a strict guarantee of data protection.

## Discussion

### Principal Findings

Despite the benefit that current maternal education can bring to women, new tools are needed to increase its effectiveness and reach. Proposals for renewal arise and are permanently evaluated [39-41]. Digital tools have proven to be useful, and at times like the present, they have been revealed as essential, which is why it is necessary for health care workers to explore new areas of care and support for women [38].

The creation of a collaborative environment, the involvement of the affected population, and the application of international standards to aid patient decision-making have resulted in a proposal for a digital tool that not only reports back, as is usual with these tools, but also interacts with the woman, allowing her to self-assess her health needs in order to take decisions, self-manage, and respond to these needs.

The resources included in our prototype are, in addition to exhaustive information, communication systems between peers and with professionals, validated self-assessment instruments, follow-up plans based on clinical practice guidelines, and connection with health and social resources in the community. The website will allow women to access their clinical data, and its use will be enhanced by making it accessible from any type of device, whether tablet, mobile, or computer, safely.

This is a prototype and a proposed tool. The next step is to run a pilot test in order to find out its clinical effectiveness, usability, and acceptability by users and professionals, and to see its impact on the health system.

### Comparison With Prior Work

Interventions are usually designed, and then, their degree of implementation in clinical practice, their acceptability, and their usefulness are evaluated. A study related to the acceptance of telemedicine [42] concluded that it is essential to take into account the individual characteristics of the end user in the design of the tools and their usefulness as perceived by professionals when handling them in clinical practice. These conclusions are in line with our tool design methodology and with models such as the CFIR (Consolidated Framework for Implementation Research) [43] or the TDF (Theoretical Domains Framework) [44], and the IPDAS methodology [31], which have guided our work. In addition, the internal and external contexts where it will be implemented and the implementation process itself has also been taken into account. We have not found any studies on websites aimed at pregnant or postpartum women that describe, step by step, how the web design was carried out to facilitate subsequent implementation.

Regarding content, in general, websites dedicated to pregnancy offer information on the process and resources available for care, focusing on specific aspects, such as suitable weight gain, giving up toxins, and increasing vaccination rates [45]. In the postpartum period, they also address specific problems, such as the possibility of screening for anxiety and depression [46], which is then followed up with psychoeducational interventions, peer support, and psychological therapy. Very few tools offer comprehensive care for women during pregnancy and in the postpartum period, with the exception of the NHS website.

### Limitations

The fact that we looked at the specific needs of pregnant and postpartum women in Basque Country plays in favor of the adaptability of the tool to our context; however, it might not be generalizable to other populations with different characteristics, thus requiring adaptation when extrapolating it to other contexts.

The maintenance of this type of tool requires the involvement of professionals and the organization in which they work, that is, the involvement of health service managers. The job stability of professionals in the public health system favors the continuity of the tool, as well as their participation in its design. However, pressure on health care resulting from COVID-19 may constitute a barrier to its implementation and maintenance. The involvement of the organization might also be diminished by other priorities at this difficult moment.

### Conclusions

Although it is true that some health websites are not widely used or are declining in use [41], the “EMAeHealth” platform we propose has shown, since its inception, some characteristics that would facilitate both its adoption and maintenance, as well as its effectiveness in increasing the quality of the care provided [31].

First, its design and development are based on needs expressed by users and health professionals, which will probably improve its implementation [47]. Its flexibility and adaptability will also facilitate its use.

Second, it is proposed as a resource that would be part of the public system. Websites from official organizations have proven to be more rigorous and reliable [36]. Additionally, they can eliminate any hint of conflict of interest that could occur on privately financed websites and favor more universal use, eliminating possible differences based on the socioeconomic level [48,49]. This tool makes it possible to link support for shared decision-making with the use of clinical practice guidelines as a source of evidence and as a basis for recommendations on care or self-care, which would boost acceptance and adoption of the latter by professionals.

Third, it is a customized tool. Among all patients generally, in addition to their physical and mental well-being, health care has to attend to patients’ values, beliefs, or capacities [50], but this is a particularly substantial part of attention in maternity. Pregnancy is a physiological stage in principle, but also a time when self-perception, parenting styles, and other elements are the most relevant variables, and in which other actors, such as partners and family members, with their own customs, convictions, or even ideologies (cultural and social baggage), are directly involved.

Naturally, the website described here should not be seen as a substitute for personal attention [2,31]. Research shows that, in addition to regularly consulting the internet, women go to midwives and consider them their best source of information [12,13,51]. Although useful, these web tools are not sufficient to meet all the needs of women at this stage [52]. Their full potential would be achieved as support options, serving women who wish to take a more active role in managing their health.

In conclusion, health professionals must participate in the adaptation of new technologies in daily practice [53], which attend to the needs of the population. Involving the public in the design phase will facilitate the implementation of EMAeHealth and similar resources as generators of a higher quality of life for women, without increasing costs in health systems.

## Abbreviations

IPDAS: International Patient Decision Aid Standards
SDM: shared decision-making
